# Association between Outdoor Fungal Concentrations during Winter and Pulmonary Function in Children with and without Asthma

**DOI:** 10.3390/ijerph13050452

**Published:** 2016-04-28

**Authors:** Masanari Watanabe, Hisashi Noma, Jun Kurai, Degejirihu Hantan, Naoto Burioka, Sachiko Nakamoto, Hiroyuki Sano, Jumpei Taniguchi, Eiji Shimizu

**Affiliations:** 1 Department of Respiratory Medicine and Rheumatology, Faculty of Medicine, Tottori University, 36-1 Nishi-Cho, Yonago 683-8504, Japan; junkurajun@gmail.com (J.K.); degujirefu@med.tottori-u.ac.jp (D.H.); k2x3dp@gmail.com (J.T.); eiji@med.tottori-u.ac.jp (E.S.); 2 Department of Data Science, Institute of Statistical Mathematics, 10-3 Midori-Cho, Tachikawa, Tokyo 190-8562, Japan; noma@ism.ac.jp; 3 Division of School of Health Science, Department of Pathobiological Science and Technology, Faculty of Medicine, Tottori University, 36-1 Nishi-Cho, Yonago 683-8504, Japan; burioka@med.tottori-u.ac.jp (N.B.); naka827@grape.med.tottori-u.ac.jp (S.N.); 4 Department of Respiratory Medicine and Allergology, Faculty of Medicine, Kinki University, Ohnohigashi 377-2, Osakasayama 589-0014, Japan; hsano@med.kindai.ac.jp

**Keywords:** children, fungi, outdoor, particulate matter, pulmonary function

## Abstract

Outdoor fungi are important components of airborne particulate matter (PM). However, the associations between pulmonary function and outdoor fungi are less well known compared to other airborne PM constituents. The objective of this study was to investigate the association between outdoor fungi and pulmonary function in children. Morning peak expiratory flow (PEF) rates were measured daily in 339 schoolchildren (including 36 with asthma), aged 10 to 12, 2 to 27 February 2015. Airborne PM was collected on filters, using a high volume air sampler, each day during the study period. The daily concentration of outdoor fungi-associated PM was calculated using a culture-based method. A linear mixed model was used to estimate the association between PEF values and daily concentrations of outdoor fungi, and the daily levels of suspended PM (SPM) and PM ≤ 2.5 μm (PM_2.5_). An increase in the interquartile range (46.2 CFU/m^3^) for outdoor fungal concentration led to PEF changes of −1.18 L/min (95% confidence interval, −2.27 to −0.08) in all children, 1.22 L/min (−2.96 to 5.41) in children without asthma, and −1.44 L/min (−2.57 to −0.32) in children with asthma. Outdoor fungi showed a significant negative correlation with PM_2.5_ levels (r = −0.4, *p* = 0.04), but not with SPM (r = ‒0.3, *p* = 0.10) levels. Outdoor fungi may be associated with pulmonary dysfunction in children. Furthermore, children with asthma may show greater pulmonary dysfunction than those without asthma.

## 1. Introduction

A number of epidemiological studies have demonstrated an association between airborne particulate matter (PM) and respiratory disorders [1,2]. Similarly, several reviews and meta–analyses have demonstrated the effects of airborne PM on pulmonary function in children [3,4,5,6]. However, the results of each study showed considerable variability. Airborne PM is a mixture of solid particles and liquid droplets originating from various natural and anthropogenic sources [7]. The constituents and components of airborne PM differ according to area and season [8,9]. The discordance in the results of various studies that have investigated the association between airborne PM and pulmonary function may be attributable to the disparity in the composition of airborne PM [3,4,5,6]. Additionally, to date, it remains unclear which constituents and components of airborne PM are most responsible for pulmonary dysfunction.

Bioaerosols are one component of airborne PM; fungi are included in bioaerosols [10]. Airborne PM is usually categorized according to particle size as PM_10_ or PM_2.5_, representing median aerodynamic diameters smaller than 10 μm or 2.5 μm, respectively [11]. Inhaled airborne PM can affect different parts of the respiratory tract, depending on particulate size [12]. While coarse PM (PM_2.5–10_) is deposited primarily in the bronchus, fine PM (PM_2.5_) is more likely to disperse deeper into the tracheobronchial and alveolar regions, where it is deposited [10,13]. Fungi are included in both coarse and fine PM [13], and can thus be responsible for various respiratory disorders, including asthma, allergic bronchial pulmonary mycosis, and hypersensitivity pneumonitis [14]. Materials of biological origin contribute as much as 25% to the atmospheric aerosol [15]. However, biological PM in the outdoor atmosphere has received less attention than other aerosol particles, such as sulfates and mineral dust [10]. In particular, relatively little is known about the association between short term exposure to outdoor fungi (as components of airborne PM) and pulmonary function in children.

The purpose of this study was to investigate the association between pulmonary function in schoolchildren and the daily concentrations of fungi present in PM. Airborne fungal spores are an important risk factor for asthma and can trigger asthma exacerbations, particularly in children [16,17]. The effects of exposure to fungi on pulmonary function may differ between children with and without asthma. Therefore, this study aimed to assess the difference in the effect of airborne fungi on pulmonary function in children with and without asthma.

## 2. Materials and Methods 

### 2.1. Study Design

In this panel study, morning peak expiratory flow (PEF) rates were measured in schoolchildren daily 2 to 27 February 2015. The study was conducted in Matsue, the capital city of the Shimane Prefecture, in southwest Japan. Matsue has a population of approximately 200,000 inhabitants and covers a geographical area of 530.2 km². A total of 345 children, aged 10 to 12 years, participated in the study. They were enrolled from four (of a total of 35) elementary schools. The four schools were within 10 km of each other, and all participating children lived within a 1 km radius of their school.

Patient data—including sex, age, height, weight, and the presence of asthma, allergic rhinitis, allergic conjunctivitis, atopic dermatitis, and food allergies—were recorded in February 2015. The participants were considered to have asthma if they met any of the following criteria during the preceding 12 months: diagnosis of asthma by a pediatrician, presence of wheezing, use of asthma medication, and/or a visit to a hospital for asthma. Furthermore, they were considered to have allergic rhinitis, allergic conjunctivitis, atopic dermatitis, and/or food allergy if they met any of the following criteria during the preceding 12 months: diagnosis of any of these conditions by a pediatrician, use of medication for any of these conditions, and/or a visit to a hospital for any of these conditions. 

The study was approved by the institutional ethics committee (Ethics Committee of the Faculty of Medicine, Tottori University: Approval Number 2473). The study was also approved by the Matsue City Board of Education and the Parent Teacher Association of each elementary school involved in the study. The children and their parents provided written consent after receiving information about the study from their teachers.

### 2.2. Recording of Daily Morning Peak Expiratory Flow

The participating children and their teachers were taught how to measure PEF before the start of the study. From 2 to 27 February 2015, all children measured their PEF every morning using a peak flow meter (Mini-Wright, Harlow, UK, American Thoracic Society Scale). The children recorded their best PEF value after three attempts between the hours of 8:00 A.M. and 9:00 A.M. All children walked to school and were potentially exposed to air pollutants during their commute.

### 2.3. Measurement of Air Pollutant Levels 

Suspended particulate matter (SPM) is defined under the National Air Quality Standard as any particle with a diameter smaller than 10 μm with a 100% cut-off [18]. In Japan, the Japanese Ministry of the Environment monitors the levels of SPM instead of PM_10_. The concentrations of SPM, PM_2.5_, sulfur dioxide (SO_2_), nitrogen dioxide (NO_2_), and ozone are monitored by the Japanese Ministry of the Environment in Matsue City, which is the sole monitoring station. Meteorological variables in Matsue City, such as daily temperature, relative humidity, and atmospheric pressure, were also measured by the Japan Meteorological Agency. The data for Matsue City obtained from the Japanese Ministry of the Environment and the Japan Meteorological Agency were used to examine the associations between changes in PEF and air pollutant levels. Daily average levels of air pollutants, such as SPM, PM_2.5_, SO_2_, NO_2_, and ozone, were calculated from 6:00 A.M. to 5:00 A.M. the following day.

### 2.4. Calculation of Daily Concentrations of Fungi-Associated PM

The daily concentration of outdoor fungi-associated PM was calculated using culture-based methods [10,19]. In Chuo Elementary School, one of schools participating in this study, total suspended particles were collected on 20 × 25 cm quartz filters (2500QAT-UP; Tokyo Dylec, Tokyo, Japan) at a flow rate of 1000 L/min using a high-volume air sampler (HV-1000R; Shibata, Tokyo, Japan) for 23 h—from 7:00 A.M. to 6:00 A.M. the following day. This sampling took place from 1 February to 28 February 2015. After sampling, a 4-cm^2^ filter was detached and PM was extracted with 4 mL of distilled, deionized water. For fungal culture, 500 μL of the 4 mL PM suspension was spread onto Sabouraud agar culture in 90 mm diameter dishes and incubated for 5 days at 28 °C. After 5 days, the growing colonies were counted and the mean value of five dishes was calculated. The daily concentrations of outdoor fungi-associated PM were expressed as colony forming units per cubic meter of air (CFU/m^3^).

### 2.5. Statistical Analysis

Linear mixed models that accounted for correlations in repeated measurements within an individual were used to estimate the effect of exposure to SPM, PM_2.5_, and fungi-associated PM on the daily PEF of children [20,21]. The linear mixed models included a random intercept, individual characteristics (gender, age, height, weight, asthma, allergic rhinitis, allergic conjunctivitis, atopic dermatitis, and food allergy); meteorological variables, such as daily temperature, relative humidity, and atmospheric pressure; gaseous air pollutants (SO_2_, NO_2_, and ozone); and the exposure variables. Linear mixed model analyses were performed using R statistical software (version 3.2.2, R Foundation for Statistical Computing, Vienna, Austria). Estimates were given as the absolute difference in PEF values per interquartile range (IQR) change in exposure, with 95% confidence intervals (CIs). Multiple imputation was used to deal with missing data, as this method adequately addresses the uncertainty around the prediction of missing values [22]. The two-pollutant model was applied to different combinations of pollutants to assess the stability of the effects of fungal concentrations on PEF after adjustment for individual characteristics (age, gender, height, weight, and presence of asthma, allergic rhinitis, allergic conjunctivitis, atopic dermatitis, and food allergies), and meteorological variables (temperature, humidity, and atmospheric pressure). Differences between children with and without asthma were evaluated by the ordinary interaction tests, Wald tests for differences of the regression coefficients between the two groups. Associations between fungal concentrations and SPM and PM_2.5_ levels were assessed by linear regression analysis, using SPSS statistical software (Japanese ver. 21.0 for Windows; IBM Japan, Tokyo, Japan). All *p*-values were two-sided, with a significance level of 0.05.

## 3. Results

### 3.1. Participant Characteristics

Of the 345 children recruited, six were excluded because they failed to maintain a daily record of their PEF rates. The characteristics of the remaining 339 children, including those with asthma (n = 36) and without asthma (n = 303), are shown in Table 1. Data were missing for sex, age, height, and body weight for 2, 3, 6, and 8 children without asthma, respectively.

### 3.2. SPM and PM_2.5_ Levels and the Relationship between SPM, PM_2.5_, and Fungi

**Figure 1:**
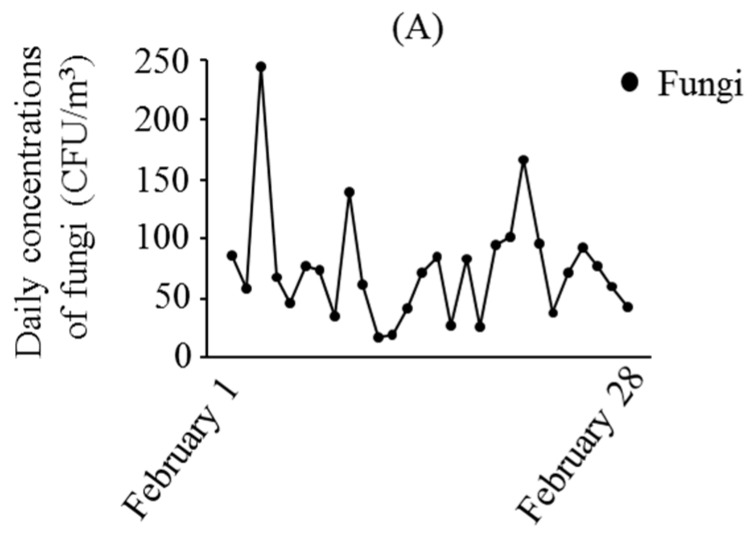

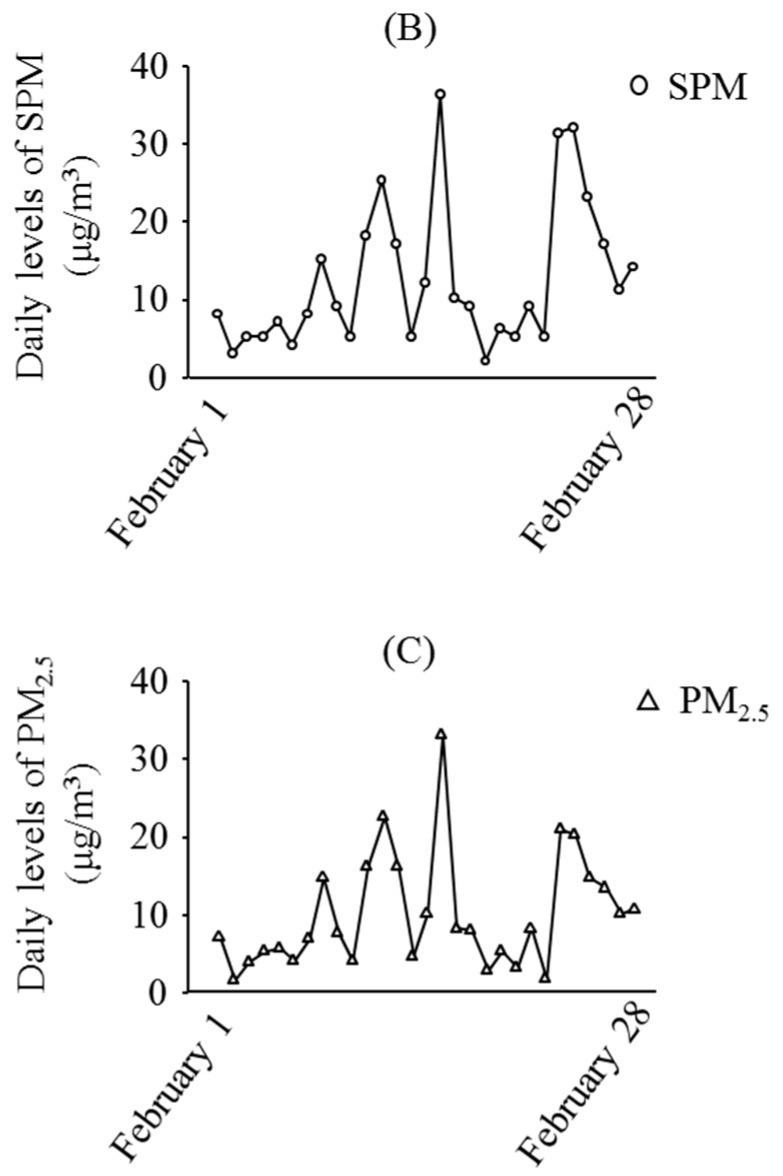
Daily concentrations of fungi (**A**); average levels of suspended particulate matter (SPM) (**B**); and particulate matter smaller than 2.5 μm in diameter (PM_2.5_) (**C**) 1 to 28 February 2015.

**Figure 2:**
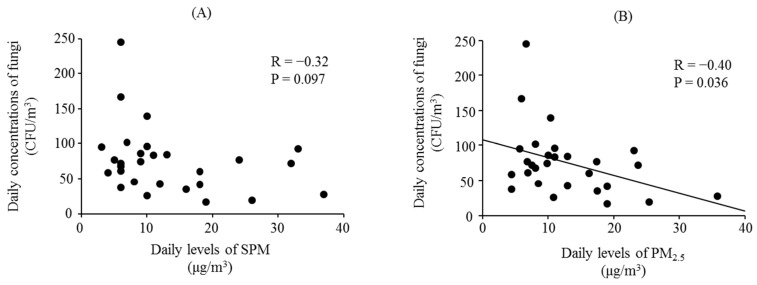
Associations between daily concentrations of fungi and the daily levels of (**A**) suspended particulate matter (SPM) and (**B**) particulate matter less than 2.5 μm (PM_2.5_). Associations between fungal concentrations and daily levels of SPM and PM_2.5_ levels were assessed by linear regression analyses.

Figure 1 presents the daily levels of SPM, PM_2.5_, and concentrations of fungi, 1 February to 28 February 2015. Figure 2 shows the associations between the daily fungal concentrations and the daily averages of SPM and PM_2.5_ during this period. There was a significant negative relationship between daily fungal concentrations and daily averages of PM_2.5_. However, no association was found between SPM and fungal concentrations. 

### 3.3. Peak Expiratory Flow

Results of the estimated changes in PEF values for IQR increases in exposure to SPM, PM_2.5_, and fungi are presented in Table 2. The daily fungal concentrations were significantly associated with PEF values in all children and in children with asthma, with an increase of 46.2 CFU/m^3^ in fungal concentration reducing the PEF value by 1.18 L/min and 1.44 L/min, respectively. However, in children without asthma, there was no significant association between the PEF value and fungal concentration. Although there was no significant difference between children without and with asthma by the interaction test (*p* = 0.228), their subgroup estimates were clearly different. Increases of 12.0 μg/m^3^ in SPM led to changes in the PEF value of −1.36 L/min in all children, −1.38 L/min in children with asthma, and −1.14 L/min in children without asthma. There was no significant association with SPM and PEF value. Similarly, PM_2.5_ was not significantly associated with the PEF value, with a 10.0 μg/m^3^ increase in PM_2.5,_ decreasing the PEF value by −1.72 L/min in the total study cohort (−1.56 L/min in children with asthma and −3.41 L/min in children without asthma). In a two-pollutant model adjusted for SPM and PM_2.5_, the daily fungal concentrations were significantly associated with PEF values in all children and in children with asthma (Table 3).

## 4. Discussion

Several reviews and meta-analyses have demonstrated the association between short-term exposure to airborne PM and pulmonary function in children, especially those children with asthma [3,4,5,6]. Airborne PM originates from various anthropogenic and natural sources [23], including fungi [10]. However, there has been limited investigation of the association between outdoor fungi and pulmonary function. Our key finding was that increased outdoor fungal concentrations were associated with decreased pulmonary function in schoolchildren. This places a high priority on determining which constituents and components of airborne PM are responsible for health disorders [24,25]. The findings of this study suggest that outdoor fungi may play an important role in respiratory disorders induced by exposure to airborne PM.

In this study, we estimated the effects of outdoor fungi on pulmonary function stratified by the presence of asthma, and were unable to find an association with outdoor fungi and pulmonary function in children without asthma. In addition, we did not find any other study that focused on outdoor fungi or molds, non-asthmatic children, and pulmonary function. Thus, our findings cannot be directly compared to other study results. In contrast, a number of studies have reported that outdoor fungi can increase emergency department visits and hospitalization for asthma exacerbations, and can aggravate asthma symptoms [17,26,27]. This study showed that outdoor fungi were associated with pulmonary function in asthmatic children, consistent with findings by Beaumont *et al.* showing that heavy exposure to outdoor (but not indoor) fungi can decrease pulmonary function patients with asthma [28]. These findings suggest that children with asthma may show a greater decrease in pulmonary function related to outdoor fungal exposure than those without asthma. Although there was no significant difference between children with and without asthma via the interaction test, it is known that the interaction tests have low statistical power generally [29]. Thus, the non-significant result should not be interpreted as indicating no difference of exposure effects between the two populations. Notably, results of the subgroup analyses indicated a clear difference. In order to establish more reliable evidence, further studies would be required for this problem.

This study did not find any association between pulmonary function and SPM or PM_2.5_ in children with or without asthma. In contrast, our previous studies showed significant associations with pulmonary function and SPM and PM_2.5_, especially in children with asthma [30,31]. However, these discrepancies are not unexpected, given that the effects of PM on pulmonary function are not the same across studies [3,4,5,6]. In this study, although there was no significant relationship between fungal concentrations and SPM, the fungal concentrations showed a significant negative association with PM_2.5_. This result corresponds with a study by Alghamdi *et al* [10], which found an association with inverse PM_2.5_ concentrations (1/PM_2.5_) and outdoor fungi. These findings indicate that the fungal concentrations do not depend on PM mass concentrations. The associations with outdoor fungi and respiratory status—such as pulmonary function and respiratory symptoms—were greatly dependent on fungal type [32]. Additionally, PM bound with airborne pollen and fungal spores can change its biological and morphological characteristics [10,33]. The effects of airborne PM on pulmonary function in children may depend more on the composition of airborne PM, including the types of fungi present, than on its mass concentration.

Our previous study investigated the effects of Asian dust (which is sand dust emissions originating in East Asian deserts) on PEF and respiratory symptoms in asthma [34,35]. There was no significant relationship between Asian dust and PEF, despite the worsening of respiratory symptoms. Chan-Yeung *et al.* suggested that peak expiratory flow was not a sensitive parameter for detecting subtle changes in asthma compared to respiratory symptoms [36]. In the present study, the absolute changes in the PEF values may be not high when compared with the average PEF value. However, there was a significant association between pulmonary function and daily fungal concentrations. Outdoor fungi may have a greater effect on respiratory symptoms than on pulmonary function. Therefore, we should endeavor to monitor the detrimental health effects of outdoor fungi.

Nasopharyngeal dysfunction caused by allergic rhinitis is a cause of bronchoconstriction [37]. In children, atopy is associated with airway hyper-responsiveness, which can increase airway sensitivity [38]. In addition, the sex of an individual may influence the risk of wheezing and the prevalence of asthma throughout childhood [39]. Therefore, in this study as in our previous study, we adjusted the analyses for allergic diseases because the presence of allergic disease may affect the results. 

There are several limitations in this study. First, we were unable to diagnose asthma on the basis of airway hyper-responsiveness to methacholine and reversible airflow limitation. However, the prevalence of asthma among study participants was 10.6%, comparable to the reported prevalence of current wheeze in Japanese children of the same age (9.3% to 9.9%) [40]. This suggests that the diagnosis of asthma in this study may be valid. Second, the study was conducted during winter. There have been many reports concerning seasonal and monthly variations in outdoor fungi, because meteorological conditions (such as temperature, precipitation, and relative humidity) are accompanied by changes in both the strain and concentration of outdoor fungi [27,41]. Many studies have demonstrated peak concentrations of fungi during summer and early fall months, with the lowest concentrations during the winter months [41,42,43]. Thus, this study may have underestimated the effect of outdoor fungi on pulmonary function in schoolchildren. Third, the study was unable to measure individual levels of exposure to outdoor fungi and air pollution for each child. Fourth, we were unable to test sensitivity to fungal allergens in individual children. Sensitization to fungi may potentiate the association between outdoor fungi and pulmonary dysfunction. For example, in hypersensitized asthmatic children, *Alternaria* spores can induce pulmonary dysfunction [44]. *Alternaria* and *Cladosporium* spores are the most common fungal airborne particles in outdoor air [41,42,43]. However, isolated allergy to *Alternaria* spores can be detected in only a very small percentage of patients [45]. Additionally, the percentage of children sensitized to *Cladosporium*, *Aspergillus*, and *Penicillium* spores was lower than that sensitized to *Alternaria* spores [46]. Therefore, although we were unable to investigate individual sensitivity to fungal allergens, this may not have had much influence on the results. Fifth, the culture-based method used for measuring outdoor fungi was unable to detect all outdoor fungi strains, because several strains cannot grow on Sabouraud agar culture. Therefore, the study underestimated the concentrations of fungi. In future, to estimate the association between outdoor fungi and pulmonary function, we should also study outdoor fungi strains.

## 5. Conclusions

The present study found a significant association between pulmonary function in schoolchildren and daily concentrations of outdoor fungi-associated airborne PM to which the children were exposed. 

## Figures and Tables

**Table 1:** Characteristics of the 339 children included in this study.

Characteristic	All Children (n = 339)		Children with Asthma (n = 36)		Children without Asthma (n = 303)	
Boy/Girl (number)	170/167		24/12		143/158	
Age						
10-year-old	42	(12.4)	4	(11.1)	38	(12.5)
11-year-old	293	(86.4)	32	(88.9)	261	(86.1)
12-year-old	1	(0.3)	0	(0)	1	(1.4)
Height (cm)	144.6	±7.1	143.6	±6.0	144.6	±7.2
Weight (kg)	36.4	±7.1	37.1	±9.6	36.3	±6.8
Allergic diseases, excluding asthma						
Allergic rhinitis	54	(15.9)	13	(36.1)	41	(13.5)
Allergic conjunctivitis	8	(2.4)	3	(8.3)	5	(1.7)
Atopic dermatitis	26	(7.7)	9	(0.3)	19	(6.3)
Food allergies	16	(4.7)	4	(11.1)	12	(4.0)
Average peak expiratory flow (L/min)	314.2	±60.7	316.9	±63.8	313.9	±60.5

Data are shown as mean ± standard deviation or n (%). Data were missing for sex, age, height, and body weight for 2, 3, 6, and 8 children without asthma, respectively.

**Table 2:** Multivariate analysis using linear mixed models of the association between peak expiratory flow (PEF) and suspended particulate matter (SPM), particulate matter ≤2.5 μm in diameter (PM_2.5_), and fungal concentrations, by interquartile range (IQR).

**All Children (n = 339)**				
Exposure metric	IQR	Change in PEF (L/min)	95% CI	*p* value
SPM	12.0 μg/m^3^	−1.36	−2.93 to 0.22	NS
PM_2.5_	10.0 μg/m^3^	−1.72	−3.82 to 0.36	NS
Fungi	46.2 CFU/m^3^	−1.18	−2.27 to −0.08	0.036
**Children with Asthma (n = 36)**				
Exposure metric	IQR	Change in PEF (L/min)	95% CI	*p* value
SPM	12.0 μg/m^3^	−1.38	−2.99 to 0.23	NS
PM_2.5_	10.0 μg/m^3^	−1.56	−3.70 to 0.58	NS
Fungi	46.2 CFU/m^3^	−1.44	−2.57 to −0.32	0.012
**Children without Asthma (n = 303)**				
Exposure metric	IQR	Change in PEF (L/min)	95% CI	*p* value
SPM	12.0 μg/m^3^	−1.14	−7.22 to 4.96	NS
PM_2.5_	10.0 μg/m^3^	−3.41	−11.72 to 4.91	NS
Fungi	46.2 CFU/m^3^	1.22	−2.96 to 5.41	NS

CFU, colony-forming units; CI, confidence interval; NS, not significant.

**Table 3:** Estimated effects of fungal concentrations on peak expiratory flow (PEF) in a two-pollutant model after adjustment for suspended particulate matter (SPM), and particulate matter ≤2.5 μm in diameter (PM_2.5_) by interquartile range (IQR).

**All Children (n = 339)**					
Exposure metric	IQR	Adjustment	Change in PEF (L/min)	95% CI	*p* value
Fungi	46.2 CFU/m^3^	Adjusted for SPM	−1.12	−2.22 to −0.02	0.045
		Adjusted for PM_2.5_	−1.18	−2.27 to −0.08	0.035
**Children with asthma (n = 36)**					
Exposure metric	IQR	Adjustment	Change in PEF (L/min)	95% CI	*p* value
Fungi	46.2 CFU/m^3^	Adjusted for SPM	−1.39	−2.52 to −0.26	0.016
		Adjusted for PM_2.5_	−1.45	−2.57 to −0.32	0.012
**Children without asthma (n = 303)**					
Exposure metric	IQR	Adjustment	Change in PEF (L/min)	95% CI	*p* value
Fungi	46.2 CFU/m^3^	Adjusted for SPM	1.28	−2.91 to 5.48	NS
		Adjusted for PM_2.5_	1.24	−2.95 to 5.43	NS

CI, confidence interval; NS, not significant.

## Abbreviations

The following abbreviations are used in this manuscript:

CI: confidence interval
IQR: interquartile range
NO_2_: nitrogen dioxide
PM: particulate matter
PEF: peak expiratory flow
PM_10_: particulate matter smaller than 10 micrometer
PM_2.5_: particulate matter smaller than 2.5 micrometer
SD: standard deviation
SO_2_: sulfur dioxide
SPM: suspended particulate matter
